# Endothelial Netrin‐4 regulates oligodendrocyte precursor cell proliferation and differentiation via ET‐1 signaling in preterm white matter injury

**DOI:** 10.1111/bpa.70067

**Published:** 2026-01-14

**Authors:** Fuxing Dong, Weixing Yan, Qiqi Meng, Xueli Song, Bing Cheng, Yaping Liu, Yanan Liu, Chao Ren, Ruiqin Yao

**Affiliations:** ^1^ Department of Cell Biology and Neurobiology, Xuzhou Key Laboratory of Neurobiology Xuzhou Medical University Xuzhou Jiangsu Province China; ^2^ Public Experimental Research Center Xuzhou Medical University Xuzhou Jiangsu Province China; ^3^ National Demonstration Center for Experimental Basic Medical Science Education Xuzhou Medical University Xuzhou Jiangsu Province China; ^4^ Department of Human Anatomy Xuzhou Medical University Xuzhou Jiangsu Province China; ^5^ Department of Neurology, Yantai Yuhuangding Hospital Qingdao University Yantai Shandong Province China; ^6^ Shandong Provincial Key Laboratory of Neuroimmune Interaction and Regulation, Yantai Yuhuangding Hospital Qingdao University Yantai Shandong Province China

**Keywords:** endothelin‐1, Netrin‐4, oligodendrocyte precursor cells, preterm white matter injury, vascular endothelial cells

## Abstract

Perinatal hypoxia–ischemia is a leading cause of preterm white matter injury (PWMI), yet mechanisms underlying oligodendrocyte precursor cells (OPCs) dysfunction remain poorly understood. Here, we identify endothelial‐derived Netrin‐4 (*Ntn4*) as a critical regulator of OPCs proliferation and differentiation in PWMI. Developmental analysis revealed that Netrin‐4, predominantly expressed in cerebrovascular endothelial cells (ECs), peaks during postnatal myelination and correlates with OPCs marker PDGFR‐*α*. Conditional endothelial deletion of *Ntn4* in mice impaired spatial memory, induced anxiety‐like behavior, and reduced mature oligodendrocytes, accompanied by disrupted myelin ultrastructure. In a PWMI model, endothelial *Ntn4* knockout exacerbated myelination deficits and suppressed OPCs proliferation, while inducible deletion at later stages enhanced OPCs differentiation. Mechanistically, Netrin‐4‐overexpressing ECs elevated ET‐1 secretion, which promoted OPCs proliferation but inhibited differentiation via ET‐1 receptor EDNRB. Our findings reveal that endothelial Netrin‐4 is a dual regulator of OPCs dynamics in PWMI, driving proliferation via ET‐1 while impairing differentiation. Targeting the Netrin‐4/ET‐1 axis restores OPCs maturation, offering a potential strategy to mitigate myelination deficits in PWMI.

## Graphical abstract

Our findings show early increased Netrin‐4 in mice with PWMI boosts angiogenesis and OPCs proliferation. Later, high levels hinder OPCs differentiation into mature cells, impairing myelination. It may act by stimulating ET‐1 secretion.

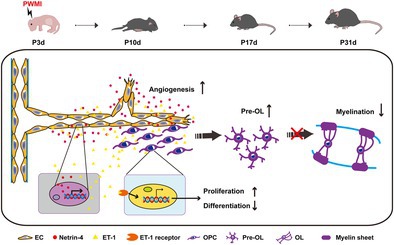

## 1 INTRODUCTION

Premature birth stands as a major and independent risk factor for neurodevelopmental disorders, posing a global health concern [1, 2]. Despite advancements in assisted reproductive technologies and neonatal medical care that have reduced mortality rates among premature infants, nearly 80% of surviving premature infants face a significantly heightened risk of neurological impairments, including cerebral palsy, and a series of effects collectively termed preterm white matter injury (PWMI) [3, 4]. This condition manifests as localized or diffuse abnormalities in myelin formation, ultimately leading to defects in gray matter connectivity [5, 6].

The neuropathological alterations associated with PWMI are complex, primarily encompassing periventricular leukomalacia and diffuse periventricular white matter injury, with the latter becoming the most prevalent form [7, 8, 9]. The primary pathological feature is the reduction or impairment of myelin formation due to perinatal hypoxia–ischemia [10, 11, 12]. Myelin dysplasia results in neurofunctional impairments, making the focus on myelin formation a crucial therapeutic target [13]. Myelin is formed by mature oligodendrocytes (OLs) differentiated from oligodendrocyte precursor cells (OPCs) [14], which are pivotal in myelin formation and play a role in insulating and providing energy support to axons [15, 16]. During the period of high incidence of PWMI, OPCs are more susceptible to damage than mature oligodendrocytes, and although they can proliferate, they struggle to differentiate into mature oligodendrocytes [12, 17, 18]. During central nervous system development, OPCs utilize a vascular endothelial scaffold originating from the subventricular zone (SVZ) for targeted migration [19], underscoring the indispensable regulatory role of the vascular niche in balancing OPC proliferation and differentiation. The adverse microenvironment in which OPCs reside may hinder their differentiation [20, 21], and abnormal development of the oligodendrocyte lineage caused by ischemia and hypoxia plays a key role in PWMI [18, 22].

The “oligovascular unit (OVU),” comprising oligodendrocyte lineage cells (OPCs, pre‐oligodendrocytes, and mature oligodendrocytes) and vascular components (endothelial cells (ECs) and pericytes) [23, 24], plays a critical role in maintaining oligodendrocyte renewal and homeostasis [23]. Factors secreted by vascular ECs promote the survival, proliferation, and migration of OPCs [23, 25]. Our previous studies have found that the PI3K/AKT/mTOR signaling pathway is involved in changes to the OVU following ischemic–hypoxic injury, and factors released after EC hypoxic injury can promote OPCs' differentiation [24, 26].

Netrin‐4, a member of the Netrin family [27, 28], is a neuroguidance factor that has garnered significant attention for its potential role in the development of the nervous system [29]. Extensive research has demonstrated that Netrin‐4 interacts with classical Netrin receptors, such as Deleted in Colorectal Cancer (DCC) and UNC5 (Unc‐5 homolog), to mediate a diverse array of biological functions, including axon guidance, angiogenesis, and tumor development [30, 31, 32]. However, recent findings by Reuten et al. have challenged this paradigm by suggesting that Netrin‐4 does not bind to known classical Netrin‐1 receptors like DCC and UNC5. Instead, it competes with the γ1 chain of Laminin, thereby exerting a crucial influence on angiogenesis [28]. Despite these insights, the precise functions and mechanisms of action of Netrin‐4 remain a subject of considerable debate within the scientific community. Through proteomics, our previous research discovered increased expression of Netrin‐4 in vascular ECs after oxygen–glucose deprivation (unpublished data). However, the expression pattern of Netrin‐4 in the central nervous system (CNS) and its impact on OPCs in PWMI remain unclear.

Endothelin‐1 (ET‐1), a potent vasoactive peptide, is implicated in CNS pathophysiology, including ischemic and inflammatory injuries [33]. It signals primarily through two G‐protein‐coupled receptors, ETA and ETB, activating downstream pathways such as protein kinase C (PKC) [34] and mitogen‐activated protein kinase [35], which regulate cellular processes like proliferation, differentiation, and inflammation [36]. In the developing brain, dysregulated ET‐1 signaling is associated with white matter damage, affecting oligodendrocyte lineage dynamics [37]. While both Netrin‐4 and ET‐1 are recognized players in neural injury and repair, whether Netrin‐4 exerts its effects specifically via modulating ET‐1 signaling in the context of PWMI remains unclear.

This study aims to investigate the regulation of Netrin‐4 secreted by cerebral vascular ECs on OPCs and its impact on myelin formation, as well as to explore its underlying mechanisms. By utilizing conditional *Ntn4* gene knockout mice and a neonatal mouse model of ischemic–hypoxic brain injury, combined with various research methods, we have conducted our studies. The findings of this research will provide new theoretical insights for the clinical treatment of PWMI.

## 2 METHODS

### 2.1 Animals

Specific‐pathogen‐free (SPF) adult male and female C57BL/6J mice (8–10 weeks old, weighing 25–30 g) were purchased from the Experimental Animal Center of Xuzhou Medical University for breeding wild‐type (WT) purposes. The day of birth was designated as postnatal day 0 (P0). The *Ntn4*‐flox mice (Strain No. T015342), Tie2‐Cre mice (Strain No. T003764), and Tie2‐CreERT2 mice (Strain No. T004737) were purchased from GemPharmatech (Nanjing, China). To generate vascular EC‐specific *Ntn4* cKO mice, Tie2‐Cre mice or Tie2‐CreERT2 mice were crossed with *Ntn4* ^f/f^ mice. We used a breeding strategy of crossing either Tie2‐Cre;*Ntn4* ^f/f^ or Tie2‐CreERT2;*Ntn4* ^f/f^ mice with *Ntn4* ^f/f^ mice, thus generating Tie2‐Cre;*Ntn4* ^f/f^ or Tie2‐CreERT2;*Ntn4* ^f/f^ offspring and *Ntn4* ^f/f^ control littermates (Supplementary Figure 1). Control littermates are written as *Ntn4* ^f/f^ in the text and figures. Genomic DNA was extracted from mouse tails and genotypes of all mice were analyzed by PCR with the according primers. All mice were maintained under specific pathogen‐free conditions with free access to food and water and housed under a 12/12‐h dark/light cycle. Data for this study were derived from a total of 203 mice (Supplementary Table 1) of both sexes. All experimental procedures of animals were approved by and performed in accordance with the Institutional Animal Care and Use Committee of Xuzhou Medical University (No. 202101A018) in compliance with National Institutes of Health standards and WMA guidelines. No human subjects were involved in this study.

### 2.2 PWMI mouse model

Neonatal mice at postnatal day 3 (P3) were anesthetized with 3% isoflurane, followed by maintenance at 1.5% isoflurane. Littermates were randomly assigned to an experimental condition. Under aseptic conditions, the right common carotid artery (CCA) was exposed through a midline neck incision, isolated, and permanently ligated at both distal and proximal ends using 6–0 surgical sutures. After a 2‐h recovery period, the pups were placed in a humidified hypoxia chamber (8% O_2_, 92% N_2_) at 37°C for 90 min to induce hypoxic–ischemic (HI) injury, as previously described [24, 38]. Sham‐operated controls underwent the same surgical procedure without CCA ligation. Following hypoxia, the pups were allowed to recover for 1 h before being returned to their dam. Mice were sacrificed at various time points ranging from 4 to 28 days post‐injury (dpi) for further analysis.

### 2.3 Administration of tamoxifen

Tamoxifen (Sigma‐Aldrich, T5648, St. Louis, MO) was dissolved in corn oil to a concentration of 20 mg/mL by shaking the solution for 3 h at 37°C. For timed induction of Cre recombination to delete *Ntn4* in vascular ECs, Tie2‐CreERT2;*Ntn4* ^f/f^ mice were intraperitoneally injected with tamoxifen at 75 mg/kg body weight once daily for three consecutive days at 13, 14, and 15 dpi.

### 2.4 BrdU administration

For the experiment on OPCs differentiation in PWMI mice, a cellular proliferation marker, 5‐Bromo‐2′‐deoxyuridine (BrdU, Sigma‐Aldrich, B5502, St. Louis, MO), was dissolved in saline to a concentration of 10 mg/mL. The mice received BrdU injection intraperitoneally (50 mg/kg) once per day at 6, 7, and 8 dpi, when OPCs are proliferating within the lesion.

### 2.5 Immunofluorescence staining

Mice were anesthetized and perfused transcardially with saline followed by cold 4% paraformaldehyde (PFA). The brains were then rapidly excised and post‐fixed in 4% PFA at 4°C overnight. Subsequently, the brains were dehydrated in 15% and 30% sucrose for 24 h each. Serial coronal sections (30 μm thickness) of the corpus callosum were obtained using a cryostat microtome (CM1950, Leica, Germany). Then, the brain sections were blocked with 10% donkey serum (diluted in PBS with 0.3% Triton X‐100) for 2 h at room temperature. Primary antibodies were applied and incubated at room temperature for 10 min, followed by overnight incubation at 4°C. The primary antibodies used in this study were anti‐Netrin‐4 (R&D, AF1132, Goat, 1:50), anti‐CD31 (Abcam, ab28364, Rabbit, 1:300), anti‐GFAP (Proteintech, 16825‐1‐AP, Rabbit, 1:500), anti‐PDGFR‐*α* (Abcam, ab203491, Rabbit, 1:500), anti‐MBP (Santa Cruz, sc‐271524, Mouse, 1:400), anti‐CC1 (Abcam, ab40778, Rabbit, 1:100), anti‐BrdU (Abcam, ab2326, Rat, 1:250, incubated with 2 M HCl at 37°C for 30 min, and rinsed with 0.1 M boric acid at room temperature for 10 min), and anti‐Ki67 (Abcam, ab279653, Mouse, 1:200). After washing with PBS, sections were incubated with fluorescent secondary antibodies for 2 h at room temperature in the dark. The secondary antibodies used in this study were IFKine™ Green Donkey anti‐Goat IgG (Abbkine, A24231, 1:200), IFKine™ Red Donkey anti‐Rabbit IgG (Abbkine, A24421, 1:200), IFKine™ Red Donkey anti‐Mouse IgG (Abbkine, A24411, 1:200), Alexa Fluor 594‐Goat anti‐Rat IgG (Abcam, ab150165, 1:500), and Alexa Fluor 488‐Goat anti‐Rabbit IgG (Abcam, ab150077, 1:500). Sections were mounted with DAPI‐containing anti‐fade medium and imaged using a laser scanning confocal microscope (Leica STELLARIS 5, Germany). Images were exported in TIFF format using Leica LAS X software.

### 2.6 Western blot (WB) assay

WB procedures were performed as previously described [39]. Briefly, total protein was extracted from mice forebrain tissues or cultured cells using RIPA lysis buffer (Beyotime Biotechnology) supplemented with 1% protease inhibitor cocktail and 1 mM PMSF, followed by quantification with the Pierce BCA Protein Assay Kit (KeyGEN BioTECH). Equal amounts of protein samples were separated by SDS‐PAGE and transferred onto nitrocellulose membranes. After blocking with 5% skim milk powder in PBST for 1 h at room temperature, membranes were incubated with primary antibodies for anti‐Netrin‐4 (Novus, NBP1‐91343, Rabbit, 1:1000), anti‐CD31 (Abcam, ab28364, Rabbit, 1:1000), anti‐GFAP (Proteintech, 16825‐1‐AP, Rabbit, 1:800), anti‐PDGFR‐*α* (Abcam, ab203491, Rabbit, 1:1000), anti‐MBP (Santa Cruz, sc‐271524, Mouse, 1:800), anti‐CC1 (Abcam, ab40778, Rabbit, 1:500), anti‐ET‐1 (Abcam, ab117757, Rabbit, 1:3000), and anti‐*β*‐actin (Abcam, ab8227, Rabbit, 1:2000) overnight at 4°C. Following incubation with the IRDye® 680RD Goat anti‐Rabbit IgG (LI‐COR Bioscience, 926–68071, 1:10000) or IRDye® 800CW Goat anti‐Mouse IgG (LI‐COR Bioscience, 926–32210, 1:20000) secondary antibodies for 2 h at room temperature, the membranes were scanned with an Odyssey scanning laser imaging system (LI‐COR Bioscience, USA), and the density of the bands was analyzed by ImageJ software (version 1.53n, NIH, Bethesda, MD, USA).

### 2.7 Morris water maze test

The water maze apparatus includes a cylindrical pool (1.2 m diameter, 40 cm height), a movable platform (8 cm diameter, 20–35 cm height), a temperature control system (22°C), and an ANY‐maze video tracking system (Stoelting Co., Illinois, USA). Mice were acclimated for 2–3 h before testing. The pool was filled with water (0.5–1 cm above the platform) containing a whitening agent (titanium dioxide solution). During training (4 times daily for 5 consecutive days), mice were placed on the platform for 30 s, then released from a random starting position. Mice unable to find the platform within 60 s were guided to it and recorded as 61 s. For testing, mice were placed on the platform for 60 s, and then released from the opposite quadrant. The system recorded entries into the target quadrant and time spent there over 60 s.

### 2.8 Open field test

The open‐field test was conducted using a testing box (50 cm × 50 cm × 30 cm) equipped with the ANY‐maze automated tracking and analysis system (Stoelting Co., Illinois, USA). Mice were acclimated in the laboratory for 2 h before testing. Each mouse was gently placed in the center of the testing box, and its movement was recorded for 5 min using the ANY‐maze software. The ANY‐maze system recorded total distance traveled, average speed, and time spent in the center and peripheral zones of the box for subsequent analysis.

### 2.9 Transmission electron microscopy

After perfusion with 4% paraformaldehyde (containing 0.5% glutaraldehyde), the mice forebrain was dissected, and the corpus callosum was trimmed into 1 mm^2^ × 2 mm blocks. These were fixed in 2.5% glutaraldehyde and 1% osmium tetroxide, followed by gradient dehydration, embedding, and ultrathin sectioning. Sections were stained with lead citrate and imaged using transmission electron microscopy (Tecnai G2 Spirit Twin, FEI, USA). The axon diameter and G‐ratio (axon diameter/total myelinated fiber diameter) were measured using ImageJ software (version 1.53n, NIH, Bethesda, MD, USA). Data were collected from at least 50 myelin sheaths per mouse, with 3 mice per group.

### 2.10 Transcriptome sequencing

Transcriptome sequencing was performed by Shanghai Majorbio Biotech (Shanghai, China). Total RNA was extracted using Trizol, with integrity and purity assessed by gel electrophoresis and NanoPhotometer. Libraries were constructed from >1 μg RNA, involving mRNA isolation, fragmentation, cDNA synthesis, adapter ligation, and PCR amplification. Purified libraries were quantified using Qubit 2.0 and sequenced on an Illumina platform. The data were analyzed on the free online platform of the Majorbio Cloud Platform (www.majorbio.com). Differential gene expression was visualized using volcano plots, displaying log2 (fold change) on the *x*‐axis and −log10 (*p*‐value) on the *y*‐axis, with upregulated and downregulated genes marked in red and green, respectively. Gene ontology (GO) enrichment analysis was performed using the DAVID online tool, with a significance threshold of *p* <0.05.

### 2.11 Real‐time quantitative PCR

Total RNA was extracted from mouse forebrain using the Trizol Reagent (Invitrogen, 15596‐018) according to the manufacturer's instructions and as previously described [40]. The cDNA was synthesized from the extracted RNA using the PrimeScript™ RT reagent kit (TaKaRa, RR037A). Real‐time quantitative PCR (qPCR) was performed on the Roche LightCycler® 480 II real‐time PCR detection system, using Taq Pro Universal SYBR qPCR Master Mix (Vazyme, Q712‐02). The relative mRNA expression levels were calculated using the 2^−ΔΔ*Ct*^ method and normalized to the endogenous control *β*‐actin mRNA levels. The primers were synthesized by Sangon Biotech (Shanghai) Co., Ltd. The primer sequences were as follows: *Edn1*, sense (5′‐GGTTGGAGGCCAT CAGCAACAGCA‐3′), antisense (5′‐AAGGACGCTGGTCCTCTGCCAGT‐3′); *Actb*, sense (5′‐AGCTGAGAGGGAAATCGTGC‐3′), antisense (5′‐TCCAGGGAGGAAGAGG ATGC‐3′).

### 2.12 Lentiviral vector production

The lentiviral vector for *Ntn4* overexpression was synthesized by Wuhan BrainVTA Co., Ltd. The selected vector, rLV‐EF1a‐3 × Flag‐*Ntn4*‐P2A‐EGFP‐PGK‐Puro‐WPRE, was constructed to contain the full‐length sequence of the mouse *Ntn4* gene, with a viral titer of ≥2e+8 TU/mL. As a control, the lentiviral vector rLV‐EF1a‐3 × Flag‐P2A‐EGFP‐PGK‐Puro‐WPRE was also prepared, exhibiting a comparable viral titer of ≥2e+8 TU/mL. Primary ECs were transfected with lentiviral vector 72 h before experiments.

### 2.13 Primary culture of brain microvascular ECs

Following hypothermic anesthesia, brains from 10‐day‐old C57BL/6J or Tie2‐CreERT2;*Ntn4* ^f/f^ mice were collected, and cerebral cortices were isolated under a stereomicroscope. The isolated cortices were gently homogenized and then mixed with an equal volume of 20% BSA solution. After centrifugation at 2500 rpm for 10 min, the purified vascular fragments were then digested with 0.25% trypsin (37°C, 60 min, with 10‐min intervals), followed by centrifugation (1200 rpm, 5 min) in DMEM/F‐12 medium (Gibco, 12400‐024, USA) with 10% fetal bovine serum (FBS, Gibco, 10091‐148, USA). The pellet was resuspended in complete medium and cultured in Poly‐L‐lysine (Sigma‐Aldrich, P5899, USA) coated T25 flasks, with complete medium replacement after 8–12 h and half‐medium changes every 48 h. Cells reached 90% confluence within 5 days for subsequent passaging. Then, ECs were divided into four groups: Control (normal WT mouse ECs), *Ntn4*‐oe (WT mouse ECs transfected with *Ntn4* overexpression lentiviral vector), *Ntn4*‐vector (WT mouse ECs transfected with empty lentiviral vector), and *Ntn4*‐cKO (Tie2‐CreERT2;*Ntn4* ^f/f^ mouse ECs).

### 2.14 Preparation of ECs conditioned medium

Following a 48‐h incubation period for the primary ECs in the groups of Control and *Ntn4*‐oe, the supernatant was harvested and subjected to concentration via a sterile 10 kDa filter tube (Millipore, UFC803096, USA). Thereafter, the concentrated supernatant was combined with standard culture medium (DMEM/F‐12 medium with 10% FBS) in a 1:1 ratio for the cultivation of primary OPCs. The conditioned medium (CM) for Control group ECs (Ctrl‐EC‐CM) and *Ntn4*‐oe group ECs (oe‐*Ntn4*‐EC‐CM) was ultimately obtained for subsequent OPCs proliferation and differentiation detection experiments.

### 2.15 Primary culture of OPCs

Primary mouse OPCs were isolated and cultured as previously described [15]. Briefly, cerebral cortices isolated from P0‐2 C57BL/6J mice under a stereomicroscope were minced in cold DMEM/F12 medium, triturated 10 times, and allowed to settle for 2 min. The supernatant was collected, centrifuged at 1200 rpm for 5 min, and then filtered through a 400‐mesh sieve. Cortical cells were seeded in Poly‐L‐lysine coated T25 flasks in the medium composed of DMEM/F12 (Gibco, 12400‐024, USA) supplemented with 10% FBS (Gibco, 10091‐148, USA) and 0.1% penicillin/streptomycin. Following 7 days of initial culture, OPCs were isolated through 18‐h shaking at 37°C and subsequently maintained for 3 days in DMEM/F12 medium supplemented with 2% B27 (Gibco, 12587‐010, USA), 10 ng/mL platelet‐derived growth factor AA (PDGF‐AA, Gibco, PHG0035, USA), and 10 ng/mL basic fibroblast growth factor (bFGF, Gibco, 13256‐029, USA) to promote proliferation.

Then, OPCs were cultured under four experimental conditions. In the Ctrl‐EC‐CM group, OPCs were incubated with conditioned medium derived from WT mouse ECs. The oe‐*Ntn4*‐EC‐CM group received conditioned medium from WT ECs overexpressing *Ntn4*. For the oe‐*Ntn4*‐EC‐CM + BQ‐788 group, OPCs were treated with the same *Ntn4*‐overexpressing conditioned medium supplemented with the ET‐1 receptor antagonist BQ‐788 (BQ‐788 sodium salt, MedChemExpress, HY‐15894, USA) at a final concentration of 1.2 nM [41]. Finally, in the BQ‐788‐only group, OPCs were cultured in standard medium containing BQ‐788 at 1.2 nM without any conditioned medium. For the differentiation of OPCs, cells were cultured in their corresponding medium for 7 days, with medium changes performed every 2 days.

### 2.16 ELISA

The levels of ET‐1 in the medium supernatant or cell lysates of mice brain microvascular ECs were determined by a sandwich ELISA kit according to the manufacturer's instructions (Nanjing Jiancheng Bioengineering Institute, H093‐1‐2, China).

### 2.17 Cerebral blood flow (CBF) measurements

To evaluate the effect of *Ntn4* on CBF in PWMI mice, we used laser speckle contrast imaging (RFLSI III, RWD Life Science, China) to measure the CBF of Tie2‐CreERT2;*Ntn4* ^f/f^ and *Ntn4* ^f/f^ mice on days 7, 14, and 28 after induction of PWMI. Briefly, mice were anesthetized using 2% isoflurane for induction, followed by maintenance at 1.5% isoflurane. Animals were positioned in a prone position with cephalic fixation. Following a midline scalp incision, the skull was exposed and cleansed with sterile saline. CBF was monitored using a high‐resolution CMOS camera system (2048 × 2048 pixels, 1 frame/s) positioned above the skull. Real‐time blood flow images were acquired and analyzed using the integrated software package, which calculated mean blood flow values for each mouse.

### 2.18 EdU labeling of OPCs

To assess cell proliferation, four groups of OPCs were cultured in 96‐well plates using their corresponding medium. The cells were incubated with 50 μM 5‐ethynyl‐2′‐deoxyuridine (EdU; Abbkine, KTA2031, China) for 2 h at 37°C. Following the labeling period, cells were fixed with 4% PFA for 30 min at room temperature. Then, cells were permeabilized with 0.5% Triton X‐100 in PBS for 10 min, followed by two washes with PBS. The incorporated EdU was then visualized using the Apollo®567 fluorescence imaging kit (Abbkine, KTA2031, China) according to the manufacturer's protocol, with a 30‐min incubation in the dark at room temperature. Nuclei were counterstained with Hoechst 33342 (5 μg/mL) for 10 min. Quantitative analysis of proliferating cells was performed by calculating the ratio of Apollo®567‐positive cells to total Hoechst 33342‐stained nuclei using a laser scanning confocal microscope (Leica STELLARIS 5, Germany).

### 2.19 Statistical analysis

Two‐tailed unpaired t test was used for comparisons between two independent groups. Multiple group comparisons were analyzed using one‐way ANOVA followed by the Sidak's or Tukey's post hoc comparison test (stated in figure legends). For comparisons of multiple factors, two‐way ANOVA with Sidak's or Tukey's post hoc comparison tests were used (stated in figure legends). Correlative analyses were performed using Pearson's correlation coefficient to evaluate the correlation between Netrin‐4 and the expression of PDGFR‐*α*, MBP, CD31 and GFAP. All quantitative data are presented as mean ± SEM. The estimate of variance was similar between all groups. Double‐blind procedures were strictly followed during the experiment, and statistical analyses were performed by an independent researcher who was also blinded to the group allocations. Statistical analyses and graphical representations of the experimental data were performed using GraphPad Prism 8.0 (GraphPad Software, Inc.). *p*‐values are annotated as follows: ns >0.05, *<0.05, ** <0.01, ***<0.001, **** <0.0001.

## 3 RESULTS

### 3.1 Hypoxia‐ischemia induced significant up‐regulation of Netrin‐4 in a PWMI mouse model

**FIGURE 1:**
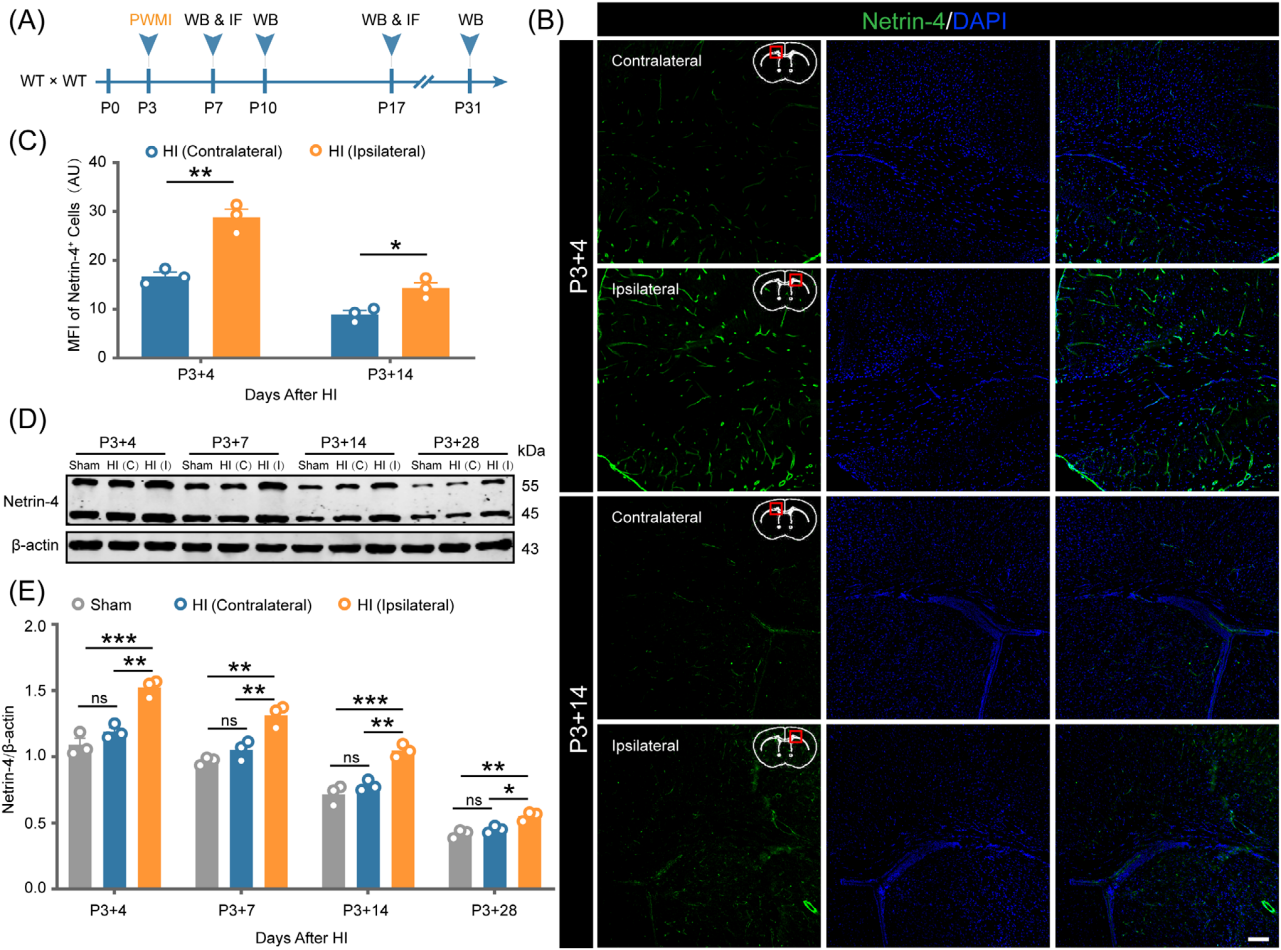
Preterm white matter injury induced significant up‐regulation of Netrin‐4. (A) Schematic depicting the experimental strategy for the detection of Netrin‐4 in mice with PWMI. (B) Representative immunofluorescence images showing temporal changes of Netrin‐4 expression in the corpus callosum of WT mice at 4 and 14 days post‐PWMI. Images depict both the ipsilateral hemisphere and contralateral hemisphere. Scale bar = 100 μm. (C) Quantitative analysis of Netrin‐4 immunofluorescence intensity. Data are expressed as mean ± SEM (*n* = 3). Statistical significance was determined by unpaired *t*‐test. *p <0.05, **p <0.01. (D) Western blot analysis of Netrin‐4 protein expression in brain tissues of WT mice at 4 and 14 days post‐PWMI injury. C: Contralateral, I: Ipsilateral. (E) Densitometric quantification of Netrin‐4 protein levels from Western blot analysis. Data are expressed as mean ± SEM (*n* = 3). Statistical analysis was performed using one‐way ANOVA followed by Tukey's post hoc test. *p <0.05, **p <0.01, ***p <0.001.

To investigate the role of Netrin‐4 in myelination disorders during mouse development, we established a PWMI model to mimic perinatal hypoxia–ischemia‐induced cerebrovascular white matter injury. Using immunofluorescence staining and WB analysis, we assessed Netrin‐4 expression in the corpus callosum of WT mice at different time points post‐PWMI (Figure 1A). Immunofluorescence (Figure 1B) revealed increased Netrin‐4 fluorescence intensity in the ipsilateral corpus callosum from 4 days post‐injury (Figure 1C, *p* <0.01), remaining significantly elevated compared to the contralateral side until 14 days (Figure 1C, *p* <0.05).

WB results (Figure 1D) showed that netrin‐4 expression peaked at day 4 post‐injury, followed by a gradual decline starting from day 7, reaching the lowest level at day 28. However, netrin‐4 expression in the ipsilateral hemisphere at each time point was significantly higher than that in the corresponding contralateral hemisphere (Figure 1E, *p* <0.01 at days 4, 7, and 14; *p* <0.05 at day 28) and sham group brains (Figure 1E, *p* <0.001 at days 4 and 14; *p* <0.01 at days 7 and 28). These findings suggest that cells in neonatal mice secrete elevated levels of Netrin‐4 during early PWMI, with sustained high expression into young adulthood. Additionally, netrin‐4 expression in sham group brains showed no difference from that in the ischemic contralateral hemisphere of the model group at each time point (Figure 1E, *p* >0.05). Therefore, we used the ischemic contralateral hemisphere of the model group instead of sham mice in all subsequent PWMI model‐related experiments in this study.

### 3.2 Netrin‐4 is primarily expressed in ECs and is associated with the development of OPCs and OLs in the postnatal mouse brain

**FIGURE 2:**
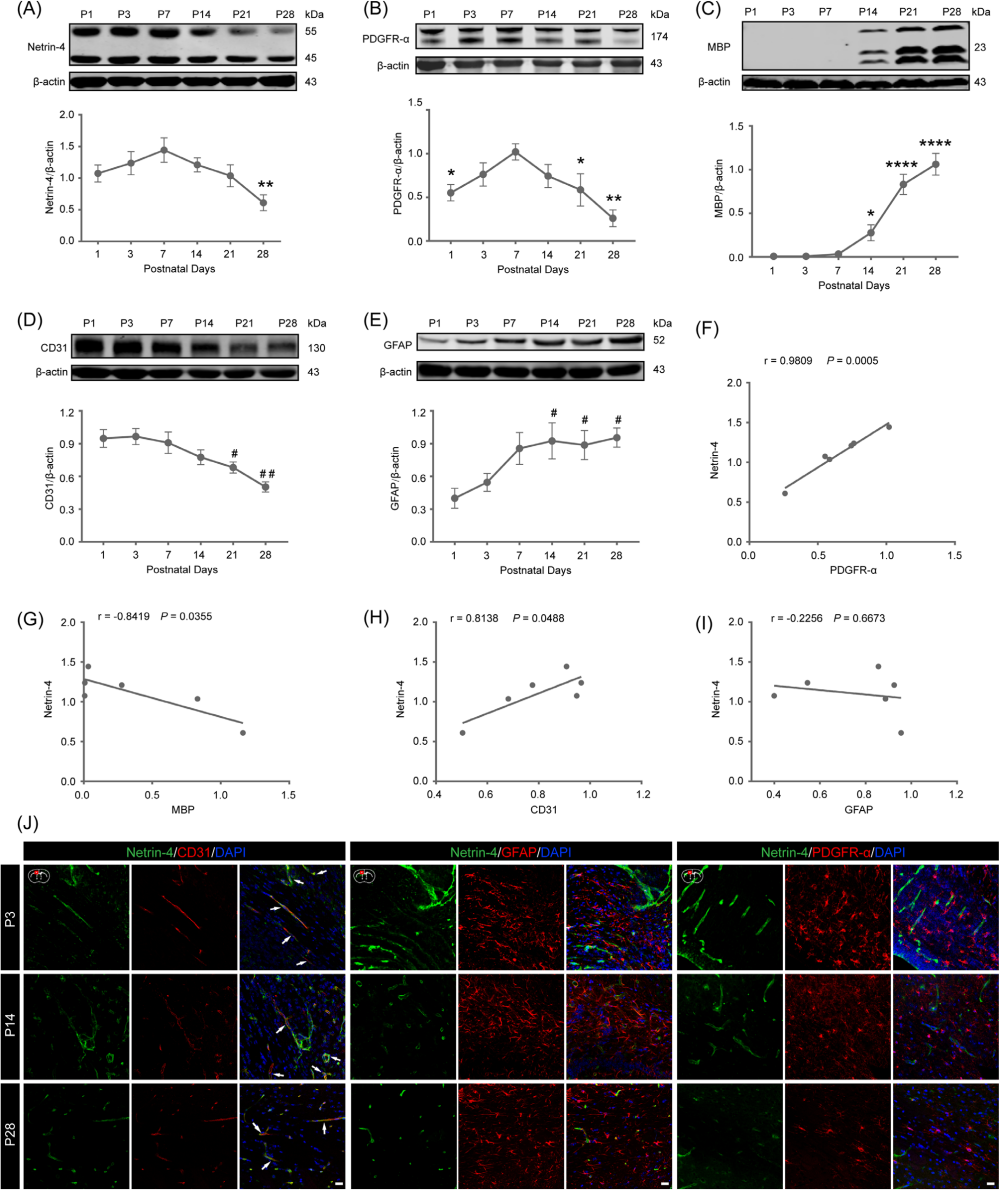
In the postnatal mouse brain, Netrin‐4 is primarily expressed in ECs and is associated with the development of OPCs and OLs. Western blot analysis of temporal expression profiles and statistical quantification of Netrin‐4 (A), PDGFR‐*α* (B), MBP (C), CD31 (D), and GFAP (E) in the brains of WT mice at postnatal days 1, 3, 7, 14, 21, and 28. Quantitative analyses of the protein expression levels were performed, with all values normalized to *β*‐actin as the internal control. Data are presented as mean ± SEM (*n* = 3). Statistical significance was determined by one‐way ANOVA followed by Sidak's multiple comparisons test. In (A)–(C) compared to the P7 group, *p <0.05, **p <0.01, ****p <0.0001. In (D) and (E), compared to the P1 group, # *p* <0.05, ## *p* <0.01. Pearson correlation analysis between Netrin‐4 expression and the expression patterns of PDGFR‐*α* (F), MBP (G), CD31 (H), and GFAP (I) in the brains of WT mice during postnatal development (days 1, 3, 7, 14, 21, and 28). Correlation coefficients (*r*) and corresponding *p*‐values are indicated for each pairwise comparison. (J) Representative immunofluorescence images demonstrating co‐localization of Netrin‐4 with CD31, GFAP, and PDGFR‐α in the corpus callosum of WT mice at postnatal days 3, 14, 21, and 28. Scale bar = 5 μm. Images were acquired using a confocal laser scanning microscope with identical acquisition parameters across samples.

We used WB to analyze changes in the expression levels of Netrin‐4 and markers for OPCs (PDGFR‐*α*), mature oligodendrocytes (MBP), vascular ECs (CD31), and astrocytes (GFAP) in the brains of normal mice on postnatal days 1, 3, 7, 14, 21, and 28. WB results revealed a dynamic expression profile for Netrin‐4, with high levels at P1, peaking at P7, and declining steadily thereafter to minimal levels by P28 (Figure 2A, *p* <0.01 compared to the P7 group). PDGFR‐*α* exhibited a similar temporal pattern, mirroring Netrin‐4's expression trajectory (Figure 2B, compared to the P7 group: *p* <0.05 at P1 and P21; *p* <0.01 at P28). In contrast, MBP expression was inversely correlated, remaining low from P1 to P7 but increasing progressively to peak at P28 (Figure 2C, compared to the P7 group: *p* <0.05 at P14; *p* <0.0001 at P21 and P28). CD31 levels were highest at P1 and gradually decreased to baseline by P28 (Figure 2D, compared to the P1 group: *p* <0.05 at P21; *p* <0.01 at P28), while GFAP expression rose steadily from P1, peaked at P14, transiently declined at P21, and increased again by P28 (Figure 2E, compared to the P1 group: *p* <0.05 at P14, P21, and P28).

Pearson correlation analysis further demonstrated a strong positive correlation between Netrin‐4 and PDGFR‐*α* (Figure 2F, *r* = 0.9809, *p* = 0.0005) and a negative correlation with MBP (Figure 2G, *r* = −0.8419, *p* = 0.0355). Netrin‐4 also positively correlated with CD31 (Figure 2H, *r* = 0.8138, *p* = 0.0488) but showed no significant association with GFAP (Figure 2I, *r* = −0.2256, *p* = 0.6673).

To elucidate the cellular source of Netrin‐4 in the neonatal mouse brain, we performed immunofluorescence co‐staining in the corpus callosum at P3, P14, and P28. The results showed that Netrin‐4 exclusively colocalized with CD31‐positive ECs, with no detectable expression in GFAP‐positive astrocytes or PDGFR‐*α*‐positive OPCs (Figure 2J). These findings indicate that vascular ECs are the primary source of Netrin‐4 in the neonatal mouse brain.

### 3.3 Vascular EC *Ntn4* deficiency exacerbates cognitive decline in young adult mice with PWMI

**FIGURE 3:**
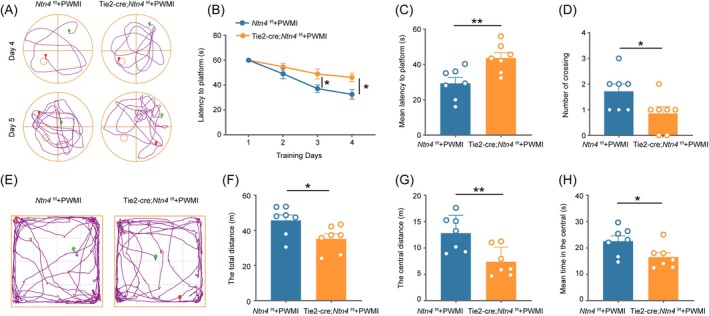
Vascular endothelial cell *Ntn4* deficiency exacerbates cognitive decline in adult mice with PWMI. (A) Representative swim paths of mice from both groups on days 4 and 5 of the Morris water maze test. (B) Mean latency to locate the platform during the first 4 days of the acquisition phase in the spatial navigation task. Data are presented as mean ± SEM (*n* = 7). Statistical analysis was performed using two‐way ANOVA followed by Sidak's post hoc test. *p <0.05. (C) Time taken to first cross the target quadrant during the probe trial on day 5 of the spatial navigation task. (D) Number of crossings over the target quadrant during the probe trial. (E) Representative trajectory plots of mice from both groups in the open field test. (F) Total distance traveled by mice in the open field test. (G) Distance traveled by mice in the center zone of the open field. (H) Time spent by mice in the center zone of the open field. In (C), (D), (F), (G) and (H), Data are presented as mean ± SEM (*n* = 7). Statistical significance was determined by unpaired *t*‐test. *p <0.05, **p <0.01.

To investigate the role of Netrin‐4 in PWMI, we generated vascular EC‐specific *Ntn4* knockout mice (Tie2‐Cre;*Ntn4* ^f/f^) and established a PWMI model. Behavioral assessments on day 28 post‐PWMI using the Morris water maze (Figure 3A) showed that *Ntn4* knockout mice exhibited significantly longer latencies to locate the platform on days 3–4 of training (Figure 3B, *p* <0.05). On day 5, after platform removal, these mice demonstrated increased latency to first cross the target quadrant (Figure 3C, *p* <0.01) and fewer platform crossings (Figure 3D, *p* <0.05), indicating impaired spatial learning and memory. In the open field test (Figure 3E), *Ntn4* knockout mice traveled shorter total distances (Figure 3F, *p* <0.05), with reduced central area exploration (Figure 3G, *p* <0.01) and less time spent in the center (Figure 3H, *p* <0.05), suggesting diminished exploratory behavior and increased anxiety‐like symptoms. These findings demonstrate that early postnatal ischemia and hypoxia in vascular EC‐specific *Ntn4* knockout mice lead to significant cognitive and behavioral deficits in young adulthood.

### 3.4 Vascular EC *Ntn4* deficiency exacerbates PWMI‐induced myelination disorder

**FIGURE 4:**
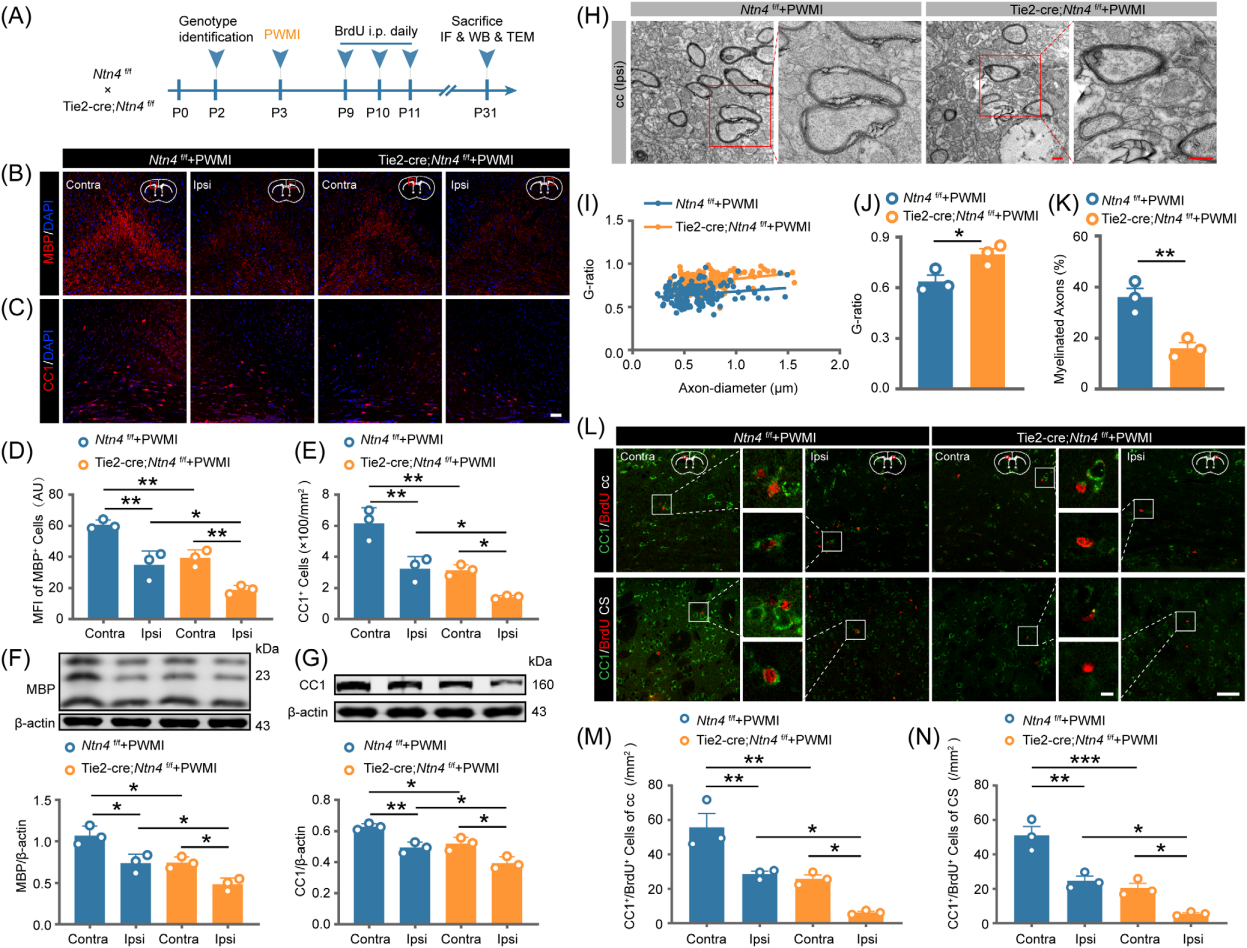
Vascular endothelial cell *Ntn4* deficiency exacerbates PWMI‐induced myelination disorder. (A) Experimental timeline of PWMI surgery, followed by IF, WB and TEM testing. Immunofluorescence staining was employed to assess changes in MBP fluorescence intensity (B) and the number of CC1‐positive cells (C) in the corpus callosum of mice 28 days post‐PWMI. Scale bar = 50 μm. Statistical analysis of the average MBP fluorescence intensity (D) and the number of CC1‐positive cells (E). Western blot analysis of MBP (F) and CC1 (G) expression and statistical analysis in the brains of mice 28 days post‐PWMI. (H) Representative ultrastructural images illustrating myelin integrity in the ipsilateral corpus callosum 28 days post‐PWMI. Scale bar = 500 nm. (I) Scatter plot of the myelin G‐ratio measurements in both groups of mice, with *n* = 50 axons analyzed per mouse. (J) Quantitative analysis of G‐ratio. (K) Quantitative analysis of the number of myelinated axons. (L) Immunofluorescence staining showing changes in CC1 and BrdU positive cells in the corpus callosum (upper panel) and corpus striatum (lower panel) 28 days post‐PWMI. Scale bar = 50 μm, zoomed images scale bar = 5 μm. Quantitative analysis of CC1 and BrdU double‐positive cells in the corpus callosum (M) and striatum (N). Data are expressed as mean ± SEM (*n* = 3). Statistical analysis was performed using two‐way ANOVA followed by Tukey's post hoc test. *p <0.05, **p <0.01, ***p <0.001. Contra: Contralateral, Ipsi: Ipsilateral.

To elucidate the role of *Ntn4* in post‐PWMI myelination, we assessed mature oligodendrocyte production in young adult mouse PWMI models (Figure 4A). The results of immunofluorescence (Figure 4B,C) showed that, compared to controls, *Ntn4* conditional knockout mice exhibited significantly reduced MBP fluorescence intensity (Figure 4D, *p* <0.05) and fewer CC1‐positive cells (CC1 is a marker for mature oligodendrocytes; Figure 4E, *p* <0.05) 28 days post‐PWMI. The ischemic ipsilateral side of knockout mice showed lower MBP intensity (Figure 4D, *p* <0.01) and CC1‐positive cell counts (Figure 4E, *p* <0.05) than the contralateral side. WB analysis confirmed reduced MBP (Figure 4F, *p* <0.05) and CC1 (Figure 4G, *p* <0.05) expression on the ischemic ipsilateral side of knockout mice compared to controls, indicating suppressed oligodendrocyte maturation in *Ntn4* knockout mice post‐PWMI. Transmission electron microscopy revealed thinner myelin sheaths (Figure 4H), increased G‐ratios (Figure 4I,J, *p* <0.05), and fewer myelinated axons (Figure 4K, *p* <0.01) in the corpus callosum of *Ntn4* knockout mice compared to controls, highlighting severe myelin defects. To assess *Ntn4*'s impact on OPCs differentiation in vivo, BrdU was injected during peak of OPCs proliferation (days 6–8 post‐PWMI). By day 28, Tie2‐Cre;*Ntn4* ^f/f^ mice showed significantly fewer CC1/BrdU double‐positive cells in the ipsilateral corpus callosum (Figure 4L,M, *p* <0.05) and corpus striatum (Figure 4L,N, *p* <0.05) compared to controls. In summary, *Ntn4* knockout exacerbates mature oligodendrocyte loss and myelination deficits in young adult PWMI models, indicating that *Ntn4* deficiency prior to injury aggravates white matter abnormalities.

### 3.5 Netrin‐4 promotes the proliferation and inhibits the differentiation of OPCs in PWMI model mice

**FIGURE 5:**
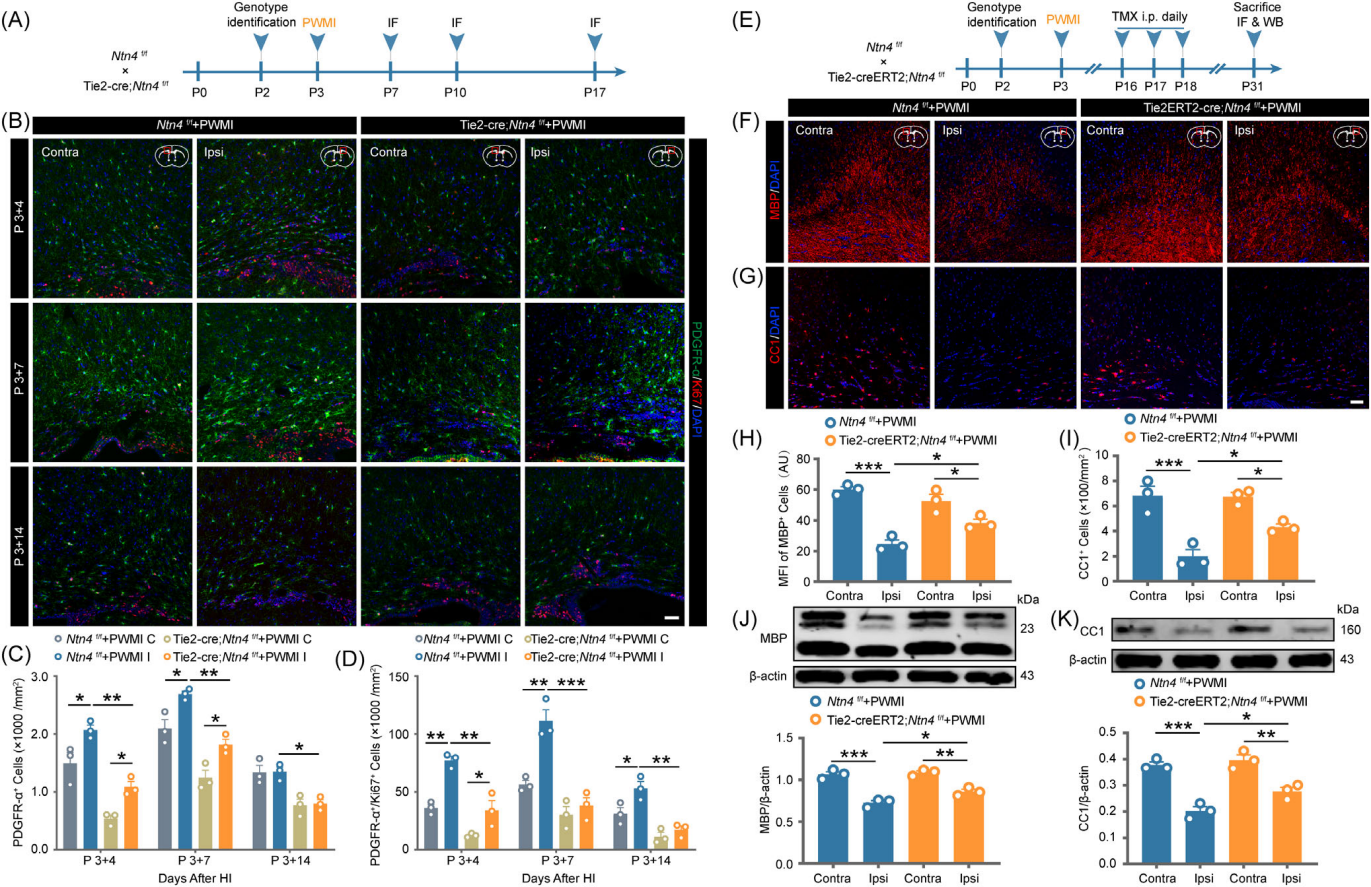
Netrin‐4 promotes the proliferation and inhibits the differentiation of OPCs in PWMI model mice. (A) Experimental timeline of the experiments in (B)–(D). (B) Immunofluorescence staining illustrating the temporal expression patterns of PDGFR‐*α* and Ki67 in the corpus callosum at post‐operative days 4, 7, and 14 following PWMI induction. Scale bar = 50 μm. (C) Quantitative analysis of PDGFR‐*α*‐positive OPCs. (D) Quantification of proliferating OPCs co‐expressing PDGFR‐*α* and Ki67. (E) Schematic representation of the experimental timeline detailing tamoxifen administration and subsequent procedures in Tie2‐creERT2;*Ntn4* ^f/f^ mice following PWMI induction. Immunofluorescent visualization of MBP (F) and CC1‐positive oligodendrocytes (G) in the corpus callosum at 28 days post‐PWMI. Scale bar = 50 μm. Quantitative assessment of MBP fluorescence intensity (H) and CC1‐positive cell density (I) in the corpus callosum. Western blot analysis and corresponding densitometric quantification of MBP (J) and CC1 (K) expression in mouse brain at 28 days post‐PWMI. All quantitative data are presented as mean ± SEM (*n* = 3). Statistical analysis was performed using two‐way ANOVA followed by Tukey's post hoc test. *p <0.05, **p <0.01, ***p <0.001. C/Contra: Contralateral, I/Ipsi: Ipsilateral.

To elucidate the role of Netrin‐4 in OPCs proliferation post‐PWMI, immunofluorescence was used to assess PDGFR‐*α* and Ki67 co‐labeling in the corpus callosum of Tie2‐Cre;*Ntn4* ^f/f^ and control mice at 4, 7, and 14 days post‐injury (Figure 5A,B). Control mice exhibited a significant increase in PDGFR‐*α* ^+^ OPCs on the ipsilateral hemisphere versus contralateral side (Figure 5C, *p* <0.05 at day 4 and day 7), with a parallel rise in Ki67^+^ OPCs (Figure 5D, *p* <0.01 at day 4 and day 7; *p* <0.05 at day 14), indicating ischemia‐induced proliferation peaking at day 7. By day 14, proliferating OPCs declined on the contralateral hemisphere but remained elevated on the ipsilateral side (Figure 5D, *p*< 0.05), suggesting impaired differentiation in late PWMI. In *Ntn4* knockout mice, ipsilateral‐side OPCs (Figure 5C, *p* <0.01 at day 4 and day 7; *p* <0.05 at day 14) and Ki67^+^ OPCs counts increased but were significantly lower than in controls (Figure 5D, *p* <0.01 at day 4 and day 14; *p* <0.001 at day 7), indicating reduced OPCs proliferation in the absence of Netrin‐4.

To evaluate Netrin‐4's role in OPCs differentiation, *Ntn4* was conditionally knocked out in Tie2‐CreERT2;*Ntn4* ^f/f^ mice via tamoxifen induction during the peak period of OPCs differentiation (days 13–15 post‐injury) (Figure 5E). At 28 days post‐injury, immunofluorescence (Figure 5F,G) revealed higher MBP intensity (Figure 5H, *p* <0.05) and increased CC1^+^ cells (Figure 5I, *p* <0.05) in the ipsilateral hemisphere of Tie2‐Cre;*Ntn4* ^f/f^ mice versus controls. WB confirmed elevated MBP (Figure 5J, *p* <0.05) and CC1 (Figure 5K, *p* <0.05) expression in knockout mice, suggesting enhanced OPCs differentiation into mature oligodendrocytes. These results imply that excessive Netrin‐4 may inhibit OPCs differentiation in late PWMI.

### 3.6 The expression of Edn1 decreased in postnatal mice brain with vascular EC *Ntn4* deficiency

**FIGURE 6:**
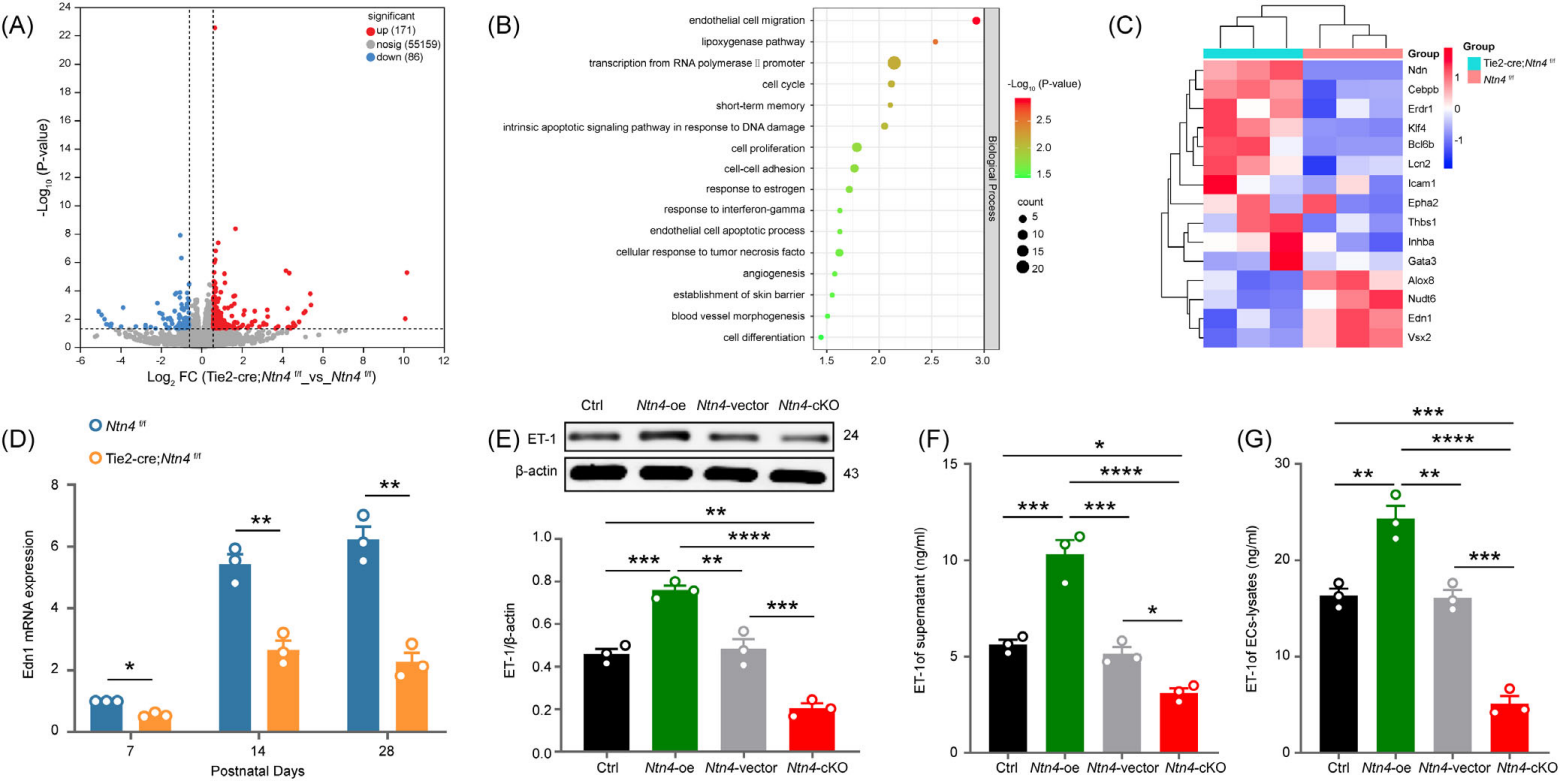
The expression of *Edn1* decreased in postnatal mice brain with vascular endothelial cell *Ntn4* deficiency. (A) Volcano plot of the results of forebrain tissue microarray analysis between the Tie2‐Cre;*Ntn4* ^f/f^ mice and *Ntn4* ^f/f^ mice at postnatal 28d (fold change ≥1.5, *p* <0.05). (B) Gene ontology (GO) enrichment analysis was performed on differentially expressed genes (DEGs) associated with biological process terms. (C) Heatmap of 15 DEGs associated with vascular functions or vascular endothelial cell biology, which were selectively identified from the GO enrichment analysis. (D) The mRNA level of *Edn1* in the forebrain at postnatal days 7, 14 and 28 by q‐PCR. Data are presented as mean ± SEM (*n* = 3). Statistical analysis was performed using two‐way ANOVA followed by Sidak's post hoc test. *p <0.05, **p <0.01. (E) Representative immunoblot bands of ET‐1 and quantification normalized to *β*‐actin in the ECs. (F) Quantification of ET‐1 levels in culture supernatants of ECs from different groups was performed using ELISA. (G) ET‐1 concentrations in cell lysates of vascular endothelial cells across various groups were determined by ELISA. In (E)–(G), data are expressed as mean ± SEM (*n* = 3). Statistical analysis was performed using one‐way ANOVA followed by Tukey's post hoc test. *p <0.05, **p <0.01, ***p <0.001, ****p <0.0001.

To investigate *Ntn4*‐mediated regulation of OPCs dynamics, we conducted transcriptomic analysis on postnatal day 28 forebrain tissues from endothelial‐specific *Ntn4* knockout (Tie2‐Cre;*Ntn4* ^f/f^) and control (*Ntn4* ^f/f^) mice. RNA sequencing identified 257 differentially expressed genes (DEGs), with 171 upregulated and 86 downregulated (Figure 6A). GO enrichment analysis highlighted significant changes in angiogenesis and vascular morphogenesis (Figure 6B). Cluster heatmap analysis revealed distinct transcriptional profiles between groups, with strong intra‐group reproducibility (Figure 6C). Among DEGs, *Edn1* (endothelin‐1, ET‐1) was a top candidate, showing reduced expression in *Ntn4*‐deficient mice, consistent with its role as a negative regulator of OPCs differentiation in demyelinating pathologies [36]. qPCR confirmed significantly lower *Edn1* mRNA levels in *Ntn4* knockout mice at multiple postnatal time points (Figure 6D, *p* <0.05 at P7; *p* <0.01 at P14 and P28).

Given that ET‐1 is primarily endothelial‐derived [42], we isolated primary brain microvascular ECs from WT and Tie2‐Cre;*Ntn4* ^f/f^ mice (7–14 days old) and generated a *Ntn4*‐overexpressing lentivirus (*Ntn4*‐oe). Cells were divided into Control, *Ntn4*‐oe, *Ntn4*‐vector, and *Ntn4*‐cKO groups. Western blotting revealed elevated ET‐1 in *Ntn4*‐oe cells (Figure 6E, *p* <0.001 vs. Ctrl) and reduced levels in *Ntn4*‐cKO cells (Figure 6E, *p* <0.01 vs. Ctrl). ELISA confirmed consistent changes in ET‐1 levels in supernatants (Figure 6F, *Ntn4*‐oe vs. Ctrl: *p* <0.001, *Ntn4‐cKO* vs. Ctrl: *p* <0.05) and cell lysates (Figure 6G, *Ntn4*‐oe vs. Ctrl: *p* <0.01, *Ntn4‐cKO* vs. Ctrl: *p* <0.001), indicating that *Ntn4* promotes ET‐1 expression and secretion in vascular ECs.

### 3.7 Angiogenesis slows down and the expression of ET‐1 decreases in PWMI mice brain with vascular EC *Ntn4* deficiency

**FIGURE 7:**
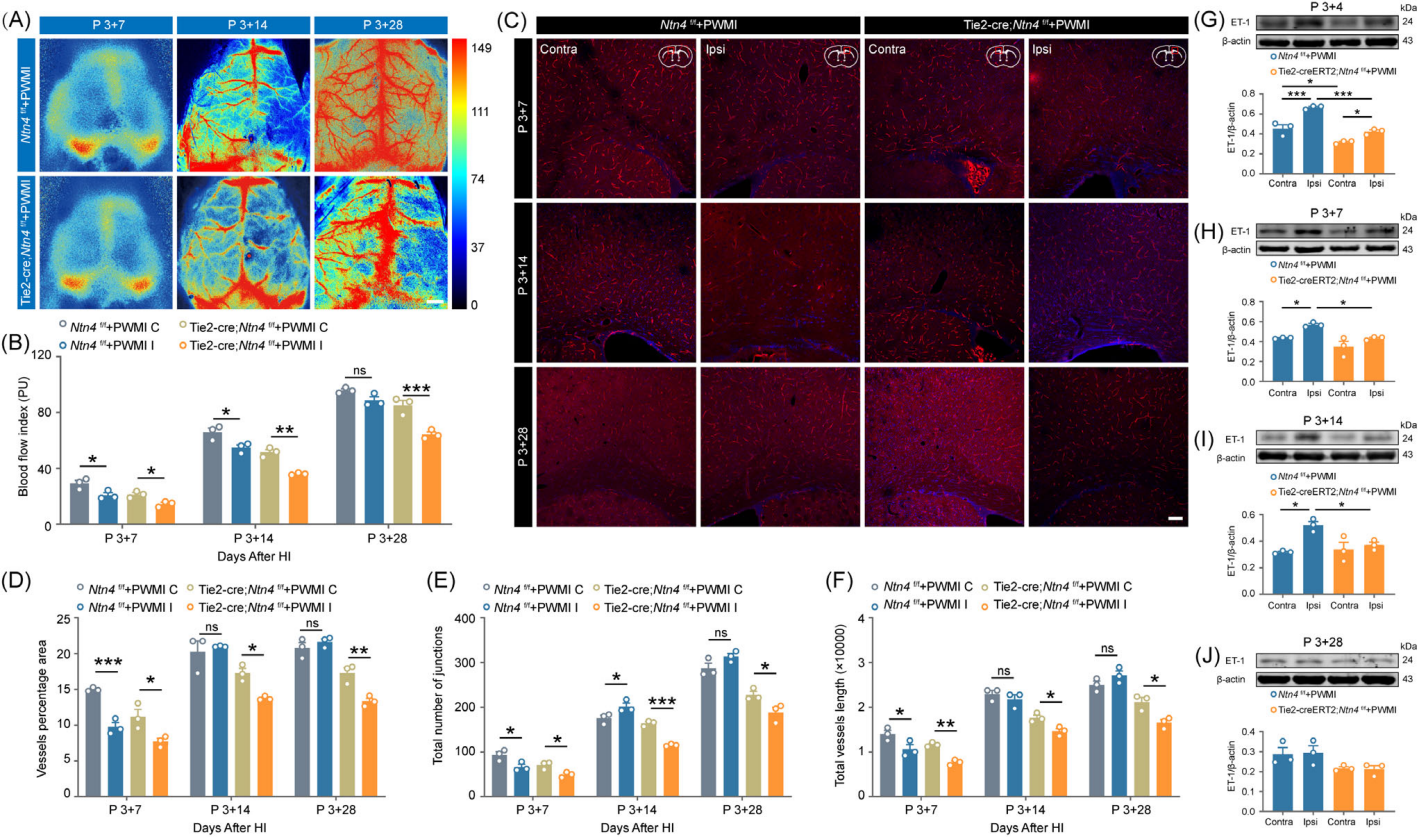
Angiogenesis slows down and the expression of ET‐1 decreases in PWMI mice brain with vascular endothelial cell *Ntn4* deficiency. (A) Representative image depicting cerebral blood flow assessed by laser Doppler flowmetry in a mouse at 7, 14, and 28 days post‐PWMI. Scale bar = 1 mm. (B) Quantitative analysis of cerebral blood flow index. (C) Representative immunofluorescence images showing CD31‐labeled blood vessels in the corpus callosum of postnatal mice following model establishment. Scale bar = 100 μm. Quantitative analysis of vascular density (D), branching points (E), and total vessel length (F) in the corpus callosum across experimental groups. Western blot analysis and quantification of ET‐1 expression in the brains of endothelial cell‐specific *Ntn4* knockout mice at 4 (G), 7 (H), 14 (I), and 28 (J) days post‐PWMI injury. Data are presented as mean ± SEM (*n* = 3). Statistical analysis was performed using two‐way ANOVA followed by Sidak's (B)–(F) or Tukey's (G)–(J) post hoc test. ns for *p* >0.05, *p <0.05, **p <0.01, ***p <0.001. C/Contra: Contralateral, I/Ipsi: Ipsilateral.

To assess the role of EC *Ntn4* in angiogenesis within a PWMI mouse model, CBF was measured at 7, 14, and 28 days post‐ischemia induction at P3 (Figure 7A). In the early phase after injury, blood flow in the ipsilateral cerebrum was significantly reduced compared to the contralateral hemisphere (Figure 7B, *p* <0.05 at day 7 and day 14 in *Ntn4* ^f/f^ mice; *p* <0.05 at day 7, and *p* <0.05 at day 14 in Tie2‐Cre;*Ntn4* ^f/f^ mice). By 28 days post‐injury, blood flow in the ipsilateral hemisphere of *Ntn4* ^f/f^ mice had recovered to a level comparable to the contralateral side (Figure 7B, *p* >0.05). In contrast, Tie2‐Cre;*Ntn4* ^f/f^ mice exhibited impaired recovery, with persistent bilateral differences at 28 days (Figure 7B, *p* <0.001), indicating that endothelial *Ntn4* ablation hinders CBF recovery. Next, immunofluorescence analysis of brain vasculature (Figure 7C) revealed reduced vessel density (Figure 7D, *p* <0.001 at day 7 in *Ntn4* ^f/f^ mice; *p* <0.05 at day 7 and day 14, *p* <0.01 at day 28 in Tie2‐Cre;*Ntn4* ^f/f^ mice), branching (Figure 7E, *p* <0.05 at day 7 and day 14 in *Ntn4* ^f/f^ mice; *p* <0.05 at day 7 and day 28, *p* <0.001 at day 14 in Tie2‐Cre;*Ntn4* ^f/f^ mice), and total length (Figure 7F, *p* <0.05 at day 7 in *Ntn4* ^f/f^ mice; *p* <0.01 at day 7, *p* <0.05 at day 14 and day 28 in Tie2‐Cre;*Ntn4* ^f/f^ mice) in the ipsilateral corpus callosum compared to the contralateral side after PWMI injury. While *Ntn4* ^f/f^ mice achieved vascular regeneration by 28 days (Figure 7D–F, *p* 0.05), Tie2‐Cre;*Ntn4* ^f/f^ mice showed impaired regeneration in the ipsilateral hemisphere, with significant bilateral differences persisting (Figure 7D, *p* <0.01; Figure 7E,F, *p* <0.05). These results suggest that endothelial *Ntn4* deletion inhibits vascular regeneration post‐ischemia. To further investigate *Ntn4*'s role, ET‐1 protein expression was analyzed by Western blotting in Tie2‐Cre;*Ntn4* ^f/f^ and control mice at 4, 7, 14, and 28 days post‐PWMI. The results demonstrated a marked elevation in ET‐1 expression on the ischemic hemisphere compared to the contralateral hemisphere in *Ntn4* ^f/f^ mice at 4, 7, and 14 days post‐PWMI injury (Figure 7G–J, *p* <0.001 at day 4; *p* <0.05 at day 7 and day 14). In contrast, Tie2‐Cre;*Ntn4* ^f/f^ mice exhibited significantly attenuated ET‐1 levels in the ischemic hemisphere at each corresponding time point relative to the control group (Figure 7G–J, *p* <0.001 at day 4; *p* <0.05 at day 7 and day 14).

### 3.8 Netrin‐4 regulates the proliferation and differentiation of OPCs through ET‐1

**FIGURE 8:**
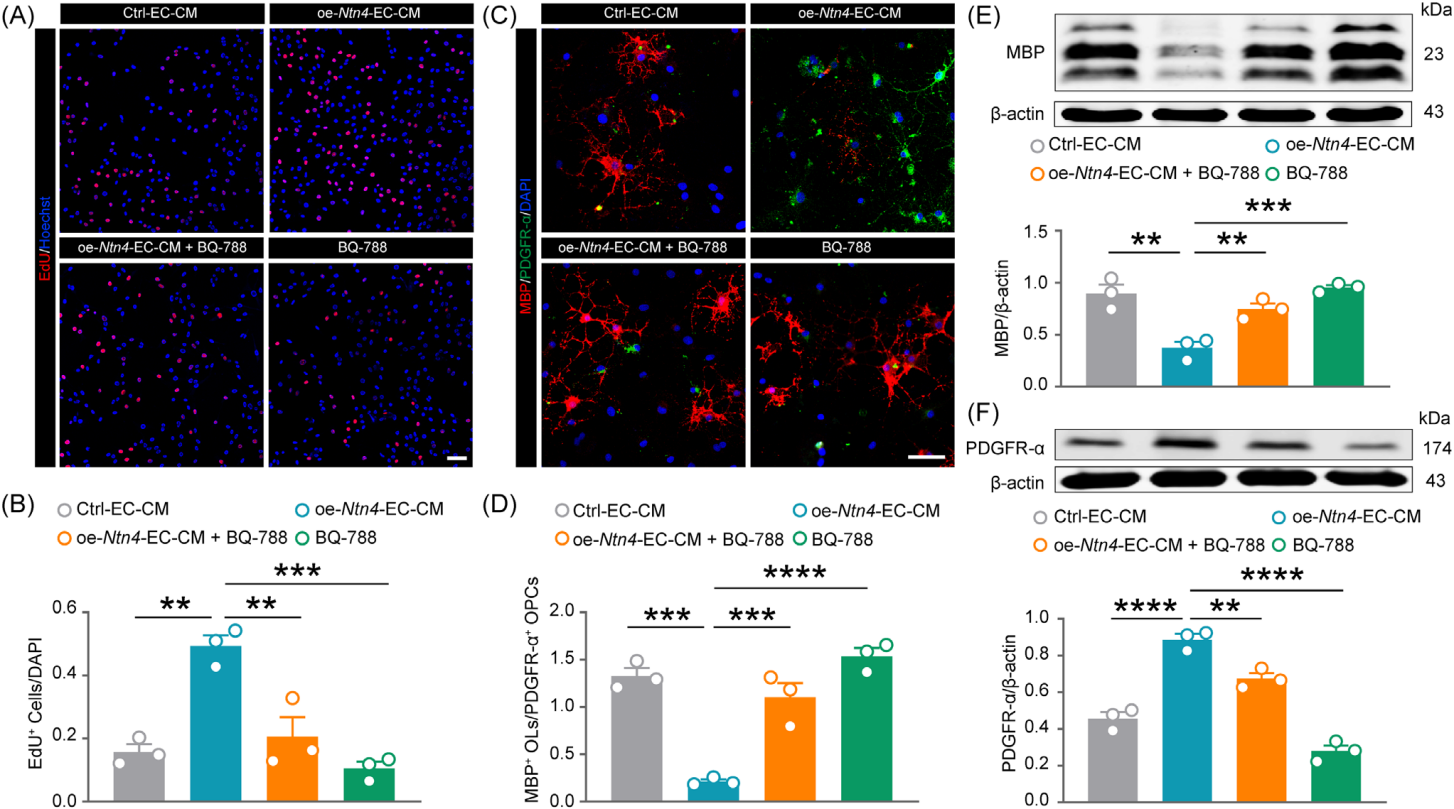
Netrin‐4 regulates the proliferation and differentiation of OPCs through ET‐1. (A) Representative image of EdU staining demonstrating proliferative changes in OPCs across groups. (B) Quantitative analysis of EdU^+^ cells. (C) Immunofluorescence staining showing the MBP and PDGFR‐*α* during OPCs differentiation. (D) Quantitative assessment of MBP^+^ OLs/PDGFR‐*α* ^+^ OPCs. Western blot analysis and quantification of MBP (E) and PDGFR‐*α* (F) expression in OPCs from different groups. Scale bars = 50 μm in (A) and (B). Data are presented as mean ± SEM (*n* = 3). Statistical analysis was performed using one‐way ANOVA followed by Tukey's post hoc test. **p <0.01, ***p <0.001, ****p <0.0001.

Increasing evidence suggests that ET‐1 promotes OPCs proliferation while inhibiting their differentiation [36, 37, 43]. To explore whether Netrin‐4 regulates OPCs via ET‐1, primary mouse OPCs were isolated and treated with conditioned medium from vascular ECs overexpressing *Ntn4* (oe‐*Ntn4*‐EC‐CM), with a protein concentration of ~10 ng/mL as determined by ELISA. OPCs proliferation was assessed using an EdU assay (Figure 8A). Compared to the Ctrl group, the proportion of EdU‐positive cells was significantly increased by oe‐*Ntn4*‐EC‐CM (Figure 8B, *p* <0.01); however, this pro‐proliferative effect was markedly attenuated in the presence of BQ‐788 (Figure 8B, *p* <0.01), whereas BQ‐788 treatment alone did not result in any significant difference relative to the Ctrl group. These results suggest that Netrin‐4 modulates OPCs proliferation via ET‐1. Next, OPCs differentiation was evaluated using immunofluorescence (Figure 8C) and Western blot. The Ctrl group exhibited more MBP‐positive oligodendrocytes and fewer PDGFR‐*α*‐positive OPCs, whereas oe‐*Ntn4*‐EC‐CM reduced MBP‐positive cells and retained more OPCs (Figure 8D, *p* <0.001). This effect was reversed by BQ‐788, which increased MBP‐positive cells and decreased OPCs (Figure 8D, *p* <0.001). BQ‐788 alone had no significant impact on differentiation. WB analysis of MBP (Figure 8E, Ctrl‐EC‐CM vs. oe‐*Ntn4*‐EC‐CM: *p* <0.01; oe‐*Ntn4*‐EC‐CM vs. oe‐*Ntn4*‐EC‐CM + BQ‐788: *p* <0.01) and PDGFR‐*α* (Figure 8F, Ctrl‐EC‐CM vs. oe‐*Ntn4*‐EC‐CM: *p* <0.0001; oe‐*Ntn4*‐EC‐CM vs. oe‐*Ntn4*‐EC‐CM + BQ‐788: *p* <0.01) expression corroborated the immunofluorescence findings. These results collectively demonstrate that Netrin‐4 modulates both proliferation and differentiation of OPCs, potentially through the ET‐1 signaling pathway.

## 4 DISCUSSION

PWMI is a significant cause of neurological deficits in premature infants, leading to long‐term cognitive and motor impairments [8]. In rodents, the developmental window spanning postnatal days 1–7 corresponds to the human gestational period of approximately 23–36 weeks [44, 45]. This phase is critical for oligodendrocyte maturation and highly vulnerable to white matter injury in preterm infants. In the present study, we modified the classic PWMI model, also referred to as the Vannucci model [46]. Specifically, in addition to the unilateral CCA ligation, we performed the procedure on postnatal day 3 in mice rather than on postnatal day 7 in rats and reduced the duration of hypoxia from 2.5 to 1.5 h [24, 38]. This milder surgical procedure substantially diminished the severe cellular death and focal injury typically associated with the classic Vannucci model [46]. Consistent with previous reports, hypoxic–ischemic insult in this model resulted in myelination deficits without inducing excessive loss of OPCs or pre‐myelinating oligodendrocytes [43, 47, 48], collectively indicating that our modified model effectively recapitulates hallmark features of preterm white matter injury. This less lethal model offers the opportunity to not only screen for signaling molecules altered during the early stages of PWMI, providing a theoretical basis for early intervention, but also to advance our understanding of the underlying molecular mechanisms, which is crucial for developing effective therapeutic strategies.

The Netrin family in vertebrates includes five subtypes (Netrins 1, 3, 4, G1a, and G1b) [29]. Among these, examination of Netrin‐4 expression by Yin et al. revealed that it is developmentally regulated in the embryonic mouse nervous system and spinal cord at stages E11.5, E14.5, and E18.5, based on in situ hybridization analysis [49]. In addition to this spatiotemporal pattern, previous studies have shown that *Ntn4* is expressed in several specific CNS regions, including the olfactory bulb, lateral olfactory tract, vomeronasal nerve, and spinal cord [49, 50], with expression levels generally higher in embryonic than in adult brains [51]. While vascular ECs secrete Netrin‐4, its expression in CNS cells, particularly those of the “oligovascular unit,” remains unclear. Using double immunofluorescence staining, we found Netrin‐4 exclusively in CD31‐positive ECs in the corpus callosum and cortex at postnatal days 3–28, with no expression in PDGFR‐*α*‐positive OPCs or GFAP‐positive astrocytes. This suggests Netrin‐4 is primarily endothelial‐derived during postnatal brain development. However, Hoang et al. reported astrocytic Netrin‐4 expression in the ischemic penumbra of adult mice after distal middle cerebral artery occlusion [52], likely due to differences in developmental stages (postnatal vs. adult) and conditions (normal vs. ischemic injury). Interestingly, consistent with our findings, no Netrin‐4‐positive astrocytes were observed in non‐ischemic regions in their study. Notably, WB and correlation analyses revealed that Netrin‐4 expression was positively correlated with OPCs' development but negatively correlated with mature oligodendrocyte development, implicating its role in postnatal white matter development. Our findings highlight that endothelial Netrin‐4 localization is particularly significant given the critical role of ECs in providing trophic support to OPCs.

Previous studies have implicated *Ntn4* in various biological processes [29, 53], including neuronal migration and axonal outgrowth [51, 54], yet its role in PWMI remains poorly understood. Here, we demonstrate that *Ntn4* is significantly upregulated in PWMI brains and dynamically regulated during injury, suggesting its involvement in white matter repair. To investigate its functional role, we generated vascular EC‐specific *Ntn4*‐deficient mice. Following PWMI induction, mice without *Ntn4* knockout showed a significant increase in OPCs in the corpus callosum, peaking at day 7 post‐injury. However, by young adulthood, these mice exhibited reduced mature oligodendrocytes, decreased myelin density, and impaired spatial learning, memory, and increased anxiety‐like behaviors, consistent with prior reports [55]. Notably, we observed exacerbated cognitive decline and myelination deficits in endothelial‐specific *Ntn4*‐deficient mice, revealing a previously unrecognized protective role for endothelial‐derived *Ntn4* in PWMI. These results imply a protective role for Netrin‐4 in PWMI, potentially via regulating OPCs' proliferation and differentiation essential for repair and recovery.

Recent studies have demonstrated that Netrin‐4 has been shown to enhance proliferation, adhesion, and migration of neural stem cells in vitro [56]. In the present study, our results indicate that Netrin‐4 promotes the proliferation of OPCs in PWMI model mice. This stimulatory effect on OPCs proliferation is crucial for maintaining the balance of the OPCs pool during the early stages of PWMI injury. Furthermore, our results demonstrate that despite the initial increase in Netrin‐4 levels and OPCs proliferation observed early after PWMI injury, elevated Netrin‐4 levels persist at 14 days post‐injury. We hypothesize that extended or aberrant expression of Netrin‐4 in the brain accounts for stalled OPCs differentiation in PWMI. To test this hypothesis, in this study, we further employed tamoxifen‐inducible conditional EC‐specific *Ntn4* knockout mice to induce *Ntn4* deletion at day 14 post‐PWMI injury, which coincides with the peak of OPCs differentiation. The results showed that in young adulthood, cKO mice exhibited higher myelin density and a greater number of mature oligodendrocytes compared to the control group, suggesting enhanced OPCs differentiation capacity. Other studies have shown that there could be a critical time window for OPCs differentiation [57, 58], and our present results demonstrate that a prolonged increase in Netrin‐4 levels is a major contributor to the OPCs differentiation failure under pathological conditions.

Here, using transcriptome sequencing analysis, we observed a decrease in *Edn1* expression in postnatal mice forebrain with vascular EC *Ntn4* deficiency. ET‐1, a small signaling peptide well‐known for its vasoconstrictor activity and systemic vasomodulatory roles in the cardiovascular system [59, 60], has recently been shown to modulate the development of neural crest cells [61], astrocytes [62], and Schwann cells [63], and is critically involved in OPCs migration, differentiation, and angiogenesis, all of which are processes essential for white matter repair [35, 36, 37]. Moreover, our findings indicate that angiogenesis is slowed and reduced ET‐1 levels in PWMI mice with *Ntn4* deficiency. These results suggest that Netrin‐4 may modulate OPCs proliferation and differentiation through the ET‐1 signaling pathway. It is interesting that recent studies have identified that OPCs express receptors for ET‐1, predominantly the B‐type receptor, EDNRB [37]. Furthermore, our in vitro results demonstrated that the addition of an EDNRB antagonist reversed the Netrin‐4‐induced proliferation and differentiation effects on OPCs. Therefore, the downregulation of ET‐1 in the absence of Netrin‐4 may contribute to the exacerbated phenotypic effects observed in our study. Our study identified ET‐1 as a downstream effector of Netrin‐4 signaling, highlighting a novel pathway by which Netrin‐4 influences OPCs development and white matter repair. This finding underscores the importance of exploring non‐classical signaling pathways in understanding the role of Netrin‐4 in neurodevelopment and repair. However, in addition to ECs, recent studies have shown that astrocytes can also secrete ET‐1, which significantly impacts OPCs proliferation and differentiation [64, 65]. Whether Netrin‐4 can regulate ET‐1 secretion by astrocytes remains to be explored in future experiments. Moreover, our study did not elucidate how Netrin‐4 regulates ET‐1 secretion or the specific signaling pathways through which ET‐1 affects OPCs. Further experiments are needed to address these questions.

This study investigates the expression, localization, and functional impact of Netrin‐4 in PWMI mice, revealing its potential involvement in white matter injury and cognitive decline. It is important to acknowledge that our experimental scope was confined to a single PWMI mouse model, which, despite being a modified and relatively mild representation, inherently cannot capture the full pathological complexity of human neonatal HIE. To broaden the applicability of our findings, future research should employ a combination of diffuse injury models (e.g., Lipopolysaccharide preconditioning with mild hypoxia–ischemia) and validate these findings using clinical samples. In addition, recent studies have identified mild malformation of cortical development with oligodendroglial hyperplasia (MOGHE) as a novel clinicopathological entity in frontal lobe epilepsy [66, 67], suggesting that netrin‐4 may participate in its pathogenesis by regulating oligodendrocyte proliferation, a potential association that warrants further investigation despite not being the focus of this study. This proposal aligns with recent studies revealing numerous myelin‐independent functions of oligodendroglia, which significantly expand their pathological implications as they have been shown to regulate neuronal development, angiogenesis, and neuroinflammatory processes [68]. Nevertheless, whether netrin‐4 contributes to these non‐myelinating functions remains an open question. Similarly, given the observed upregulation of netrin‐4 secretion by vascular ECs in mice with hindlimb ischemia [27], its role in peripheral nerve regeneration should be examined, and elucidating the effects of netrin‐4 on both myelinating and non‐myelinating Schwann cells [69] may provide novel insights into potential therapeutic strategies for nerve injury.

In summary, the present study highlights the pivotal role of Netrin‐4 in maintaining cognitive function and promoting myelination in the context of PWMI. Vascular EC *Ntn4* deficiency exacerbates PWMI‐induced cognitive decline and myelination disorders, underscoring the importance of this guidance cue in white matter repair. Our findings reveal a stage‐specific dual mechanism: during the acute phase (3–7 days post‐injury), endothelial Netrin‐4 facilitates myelination repair by promoting OPCs proliferation and physiological angiogenesis through ET‐1 signaling, while sustained expression during the chronic phase (14–28 days post‐injury) inhibits OPCs differentiation via the same pathway. This stage‐dependent regulation provides a novel therapeutic rationale for mitigating PWMI's neurodevelopmental impact. Therefore, we propose that staged pharmacological inhibition targeting the Netrin‐4/ET‐1 axis, specifically transient suppression during the recovery phase, could represent a potential therapeutic strategy for PWMI.

## AUTHOR CONTRIBUTIONS

FX.D, C.R and RQ.Y designed the study. FX.D, WX.Y, QQ.M, XL.S and B.C performed the experiments. WX.Y, XL.S, B.C, YP.L collected and analyzed the data. FX.D prepared and drafted the manuscript. YN.L, C.R and RQ.Y revised and finalized the manuscript. All authors read and approved the final paper.

## CONFLICT OF INTEREST STATEMENT

The authors have no conflicts of interest to declare.

## Supporting information


**Supplementary Table 1** Mouse quantities, experimental applications, and associated figures in this study. WT: wild‐type, PWMI: preterm white matter injury, IF: Immunofluorescence, WB: Western blot, MWM: Morris water maze, OF: Open field, TEM: Transmission electron microscopy, CBF: Cerebral blood flow, q‐PCR: Real‐time quantitative PCR, TRS: Transcriptome sequencing, EdU: EdU labeling test, ELISA: ELISA test.
**Supplementary Figure 1** Construction rationale and breeding strategies for vascular endothelial cell‐specific conditional *Ntn4* knockout mice. (A) Schematic depicting the construction rationale of Tie2‐Cre;*Ntn4* ^f/f^ mice. (B) Schematic depicting the construction rationale of Tie2‐CreERT2;*Ntn4* ^f/f^ mice. (C) The breeding strategy of Tie2‐Cre;*Ntn4* ^f/f^ or Tie2‐CreERT2;*Ntn4* ^f/f^ mice. By mating Cre heterozygous mice with *Ntn4* flox heterozygous mice, approximately one‐fourth of the offspring will possess both the Cre heterozygous and *Ntn4* flox heterozygous genotypes. When *Ntn4* flox heterozygous mice are intercrossed, around one‐fourth of the offspring will be *Ntn4* flox homozygous mice. Subsequently, mating the mice with both Cre heterozygous and *Ntn4* flox heterozygous genotypes with *Ntn4* flox homozygous mice will result in approximately one‐fourth of the offspring being Cre heterozygous and *Ntn4* flox homozygous mice, and another one‐fourth being *Ntn4* flox homozygous mice. Further mating of these two genotypes of mice will yield approximately half of the offspring as Cre heterozygous and *Ntn4* flox homozygous mice, which can serve as the *Ntn4* conditional knockout mouse, while the other half, the *Ntn4* flox homozygous littermates, will be used as controls.

## Data Availability

The data that support the findings of this study are available from the corresponding author upon reasonable request.
