# Supplementary material for: Endothelial Netrin‐4 regulates oligodendrocyte precursor cell proliferation and differentiation via ET‐1 signaling in preterm white matter injury
Source: Brain Pathol. 2026 Jan 14;36(4):e70067. doi: 10.1111/bpa.70067 (PMC13240367; doi:10.1111/bpa.70067)
Supplement: Supplementary file 1 — Supplementary Table 1 Mouse quantities, experimental applications, and associated figures in this study. WT: wild‐type, PWMI: preterm white matter injury, IF: Immunofluorescence, WB: Western blot, MWM: Morris water maze, OF: Open field, TEM: Transmission electron microscopy, CBF: Cerebral blood flow, q‐PCR: Real‐time quantitative PCR, TRS: Transcriptome sequencing, EdU: EdU labeling test, ELISA: ELISA test. Supplementary Figure 1 Construction rationale and breeding strategies for vascular endothelial cell‐specific conditional Ntn4 knockout mice. (A) Schematic depicting the construction rationale of Tie2‐Cre;Ntn4 f/f mice. (B) Schematic depicting the construction rationale of Tie2‐CreERT2;Ntn4 f/f mice. (C) The breeding strategy of Tie2‐Cre;Ntn4 f/f or Tie2‐CreERT2;Ntn4 f/f mice. By mating Cre heterozygous mice with Ntn4 flox heterozygous mice, approximately one‐fourth of the offspring will possess both the Cre heterozygous and Ntn4 flox heterozygous genotypes. When Ntn4 flox heterozygous mice are intercrossed, around one‐fourth of the offspring will be Ntn4 flox homozygous mice. Subsequently, mating the mice with both Cre heterozygous and Ntn4 flox heterozygous genotypes with Ntn4 flox homozygous mice will result in approximately one‐fourth of the offspring being Cre heterozygous and Ntn4 flox homozygous mice, and another one‐fourth being Ntn4 flox homozygous mice. Further mating of these two genotypes of mice will yield approximately half of the offspring as Cre heterozygous and Ntn4 flox homozygous mice, which can serve as the Ntn4 conditional knockout mouse, while the other half, the Ntn4 flox homozygous littermates, will be used as controls. [file BPA-36-e70067-s001.docx]

Supporting Material for

**Endothelial Netrin-4 regulates oligodendrocyte precursor cell proliferation and differentiation via ET-1 signaling in preterm white matter injury**

Fuxing Dong^1,2^ | Weixing Yan^1^ | Qiqi Meng^1^ | Xueli Song^1^ | Bing Cheng^1^ | Yaping Liu^3^ | Yanan Liu^4^ | Chao Ren^5,6^ | Ruiqin Yao^1^

^1^Department of Cell Biology and Neurobiology, Xuzhou Key Laboratory of Neurobiology, Xuzhou Medical University, Xuzhou, Jiangsu Province, China

^2^Public Experimental Research Center, Xuzhou Medical University, Xuzhou, Jiangsu Province, China

^3^National Demonstration Center for Experimental Basic Medical Science Education (Xuzhou Medical University), Xuzhou, Jiangsu Province, China

^4^Department of Human Anatomy, Xuzhou Medical University, Xuzhou, Jiangsu Province, China

^5^Department of Neurology, Yantai Yuhuangding Hospital, Qingdao University, Yantai, Shandong Province, China ^6^Shandong Provincial Key Laboratory of Neuroimmune Interaction and Regulation, Yantai Yuhuangding Hospital, Qingdao University, Yantai, Shandong Province, China

**Correspondence**

Chao Ren, Department of Neurology, Yantai Yuhuangding Hospital, Qingdao University, Yantai, Shandong Province, China

Email: renchaotg@126.com

Ruiqin Yao, Department of Cell Biology and Neurobiology, Xuzhou Key Laboratory of Neurobiology, Xuzhou Medical University, Xuzhou, Jiangsu Province, China

Email: wenxi_yao@163.com

Fuxing Dong and Weixing Yan are contributed equally to this work.

| **Mice type** | **Number** | **Experiments** | **Figures** |
| --- | --- | --- | --- |
| Normal WT P0-2 | 42 | EdU, IF, WB | Fig. 8 |
| Normal WT P1 | 3 | WB | Fig. 2A-I |
| Normal WT P3 | 6 | WB, IF | Fig. 2 |
| Normal WT P7 | 3 | WB | Fig. 2A-I |
| Normal WT P10 | 6 | WB, ELISA | Fig. 6E-G |
| Normal WT P14 | 6 | WB, IF | Fig. 2 |
| Normal WT P21 | 3 | WB | Fig. 2A-I |
| Normal WT P28 | 6 | WB, IF | Fig. 2 |
| Sham WT P3+4 | 3 | WB | Fig. 1D-E |
| Sham WT P3+7 | 3 | WB | Fig. 1D-E |
| Sham WT P3+14 | 3 | WB | Fig. 1D-E |
| Sham WT P3+28 | 3 | WB | Fig. 1D-E |
| PWMI WT P3+4 | 6 | IF, WB | Fig. 1B-E |
| PWMI WT P3+7 | 3 | WB | Fig. 1D-E |
| PWMI WT P3+14 | 6 | IF, WB | Fig. 1B-E |
| PWMI WT P3+28 | 3 | WB | Fig. 1D-E |
| Normal *Ntn4*^f/f^ P7 | 3 | q-PCR | Fig. 6D |
| Normal *Ntn4*^f/f^ P14 | 3 | q-PCR | Fig. 6D |
| Normal *Ntn4*^f/f^ P28 | 6 | TRS; q-PCR | Fig. 6A-C; Fig. 6D |
| PWMI *Ntn4*^f/f^ P3+4 | 6 | IF, WB | Fig. 5A-D; Fig. 7G |
| PWMI *Ntn4*^f/f^ P3+7 | 6 | CBF & IF, WB | Fig. 5A-D; Fig. 7 |
| PWMI *Ntn4*^f/f^ P3+14 | 6 | CBF & IF, WB | Fig. 5A-D; Fig. 7 |
| PWMI *Ntn4*^f/f^ P3+28 | 16 | MWM & OF, WB, TEM, CBF & IF | Fig. 3; Fig. 4; Fig. 5E-K; Fig. 7 |
| Normal Tie2-Cre;*Ntn4*^f/f^ P7 | 3 | q-PCR | Fig. 6D |
| Normal Tie2-Cre;*Ntn4*^f/f^ P10 | 6 | WB, ELISA | Fig. 6 E-G |
| Normal Tie2-Cre;*Ntn4*^f/f^ P14 | 3 | q-PCR | Fig. 6D |
| Normal Tie2-Cre;*Ntn4*^f/f^ P28 | 6 | TRS; q-PCR | Fig. 6A-C; Fig. 6D |
| PWMI Tie2-Cre;*Ntn4*^f/f^ P3+4 | 6 | IF, WB | Fig. 5A-D; Fig. 7G |
| PWMI Tie2-Cre;*Ntn4*^f/f^ P3+7 | 6 | CBF & IF, WB | Fig. 5A-D; Fig. 7 |
| PWMI Tie2-Cre;*Ntn4*^f/f^ P3+14 | 6 | CBF & IF, WB | Fig. 5A-D; Fig. 7 |
| PWMI Tie2-Cre;*Ntn4*^f/f^ P3+28 | 16 | MWM & OF, WB, TEM, CBF & IF | Fig. 3; Fig. 4; Fig. 5E-K; Fig. 7 |

**Supplementary Table 1** Mouse quantities, experimental applications, and associated figures in this study. WT: wild-type, PWMI: preterm white matter injury, IF: Immunofluorescence, WB: Western blot, MWM: Morris water maze, OF: Open field, TEM: Transmission electron microscopy, CBF: Cerebral blood flow, q-PCR: Real-time quantitative PCR, TRS: Transcriptome sequencing, EdU: EdU labeling test, ELISA: ELISA test.


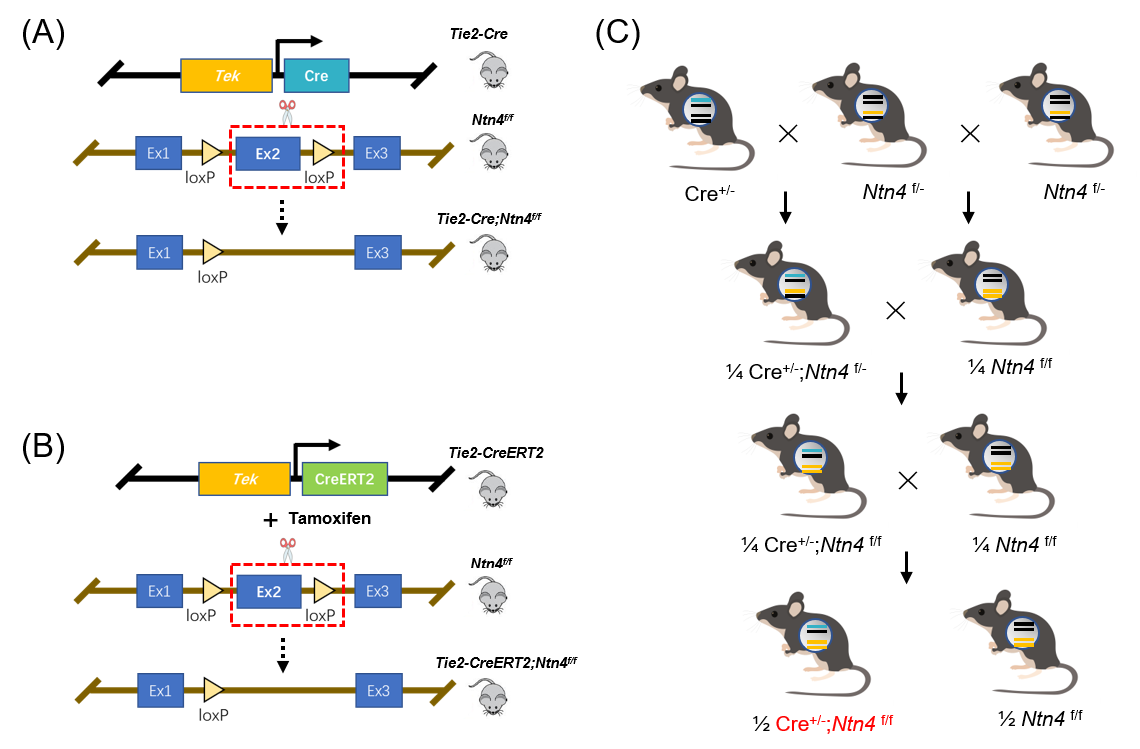


**Supplementary Figure 1** Construction rationale and breeding strategies for vascular endothelial cell-specific conditional *Ntn4* knockout mice. (A) Schematic depicting the construction rationale of Tie2-Cre;*Ntn4*^f/f^ mice. (B) Schematic depicting the construction rationale of Tie2-CreERT2;*Ntn4*^f/f^ mice. (C) The breeding strategy of Tie2-Cre;*Ntn4*^f/f^ or Tie2-CreERT2;*Ntn4*^f/f^ mice. By mating Cre heterozygous mice with *Ntn4* flox heterozygous mice, approximately one-fourth of the offspring will possess both the Cre heterozygous and *Ntn4* flox heterozygous genotypes. When *Ntn4* flox heterozygous mice are intercrossed, around one-fourth of the offspring will be Ntn4 flox homozygous mice. Subsequently, mating the mice with both Cre heterozygous and *Ntn4* flox heterozygous genotypes with *Ntn4* flox homozygous mice will result in approximately one-fourth of the offspring being Cre heterozygous and *Ntn4* flox homozygous mice, and another one-fourth being *Ntn4* flox homozygous mice. Further mating of these two genotypes of mice will yield approximately half of the offspring as Cre heterozygous and *Ntn4* flox homozygous mice, which can serve as the *Ntn4* conditional knockout mouse, while the other half, the *Ntn4* flox homozygous littermates, will be used as controls.
